# Untargeted metabolomics reveals a new mode of action of pretomanid (PA-824)

**DOI:** 10.1038/s41598-018-23110-1

**Published:** 2018-03-23

**Authors:** Rafael Baptista, David M. Fazakerley, Manfred Beckmann, Les Baillie, Luis A. J. Mur

**Affiliations:** 1 Institute of Biological, Environmental and Rural Sciences, Aberystwyth University, Penglais Campus, Aberystwyth, Wales SY23 2DA UK; 2 School of Pharmacy and Pharmaceutical Sciences, Cardiff University, Redwood Building, Cardiff, Wales CF10 3NB UK

## Abstract

Pretomanid is a promising anti-tubercular drug currently at clinical phase III, but its mechanisms of action are currently unclear. This study aimed to: (i) reveal the metabolome of *Mycobacterium smegmatis* under pretomanid treatment; (ii) compare major sources of metabolite variation in bacteria treated with pretomanid treatment and other antibiotics; and (iii) to target metabolites responsible for the killing activity of pretomanid in mycobacteria. Untargeted high-resolution metabolite profiling was carried out using flow infusion electrospray ion high resolution mass spectrometry (FIE-HRMS) to identify and quantify metabolites. The identification of key metabolites was independently confirmed by gas-chromatography time-of flight mass spectrometry (GC-tofMS) in comparison to standards. Pretomanid treatments generated a unique distinctive metabolite profile when compared to ampicillin, ethambutol, ethionamide, isoniazid, kanamycin, linezolid, rifampicin and streptomycin. Metabolites which differed significantly only with pretomanid treatment were identified and mapped on to bacterial metabolic pathways. This targeted the pentose phosphate pathway with significant accumulation seen with fructose-6-phosphate, ribose-5-phosphate and glyceraldehyde-3-phosphate. These effects were linked to the accumulation of a toxic metabolite methylglyoxal. This compound showed significant antimicrobial activity (MIC 0.65 mM) against *M. smegmatis*.

## Introduction

Tuberculosis (TB) persists as a major global threat, mostly amongst people infected with antibiotic resistant forms of *Mycobacterium tuberculosis*, the causal agent of the disease^1^. As a result, the pursuit for new anti-mycobacterials has accelerated in recent years. This search for anti-mycobacterials has identified the very promising bicyclic nitroimidazoles pretomanid (PA-824) and delamanid (OPC-67683), which are currently in clinical phase III with the Global Alliance for TB Drug Development. These have shown to be active against both replicative and hypoxic non-replicating bacilli^1,2^. This significant characteristic makes possible the relatively rapid elimination of bacteria which otherwise would require treatment for extended periods (6 to 8 months).

Studies have suggested that pretomanid could act on the mycolic acid biosynthetic pathway through depletion of ketoymycolates and the accumulation of hydroxymycolates^2^. Although this explains its anti-mycobacterial activity in replicative bacteria, it does not explain the activity of pretomanid against latent cells. Additionally, it has been shown that a des-nitroimidazole derivative from pretomanid metabolism was responsible for the generation of reactive nitrogen species and ATP depletion, which would explain its activity under anaerobic conditions^3^. This des-nitro metabolite was only identified in *Mycobacterium tuberculosis* and not in the fast-growing species *M. smegmatis*^4^. The uncertainties arising from the clear multi-target profile of pretomanid, when coupled with it clinical promise, makes the elucidation of its mode of action an urgent imperative. Understanding the interaction between pretomanid and the target bacterial metabolome could be invaluable in unequivocally defining the mechanisms of action, foreseeing future resistance mechanisms, predicting potential toxicity issues and possibly contribute to the discovery of new TB therapeutic candidates.

To address these issues the impact of pretomanid on the *M. smegmatis* metabolome is here defined using a cutting-edge untargeted metabolomic strategy^5^. *M. smegmatis* has been extensively used as model for studies for searching new anti-mycobacterials. The use of this specific strain is due to its rapid doubling time, low-pathogenicity and close genetic similarity to *M. tuberculosis*. More importantly, the metabolic pathway described in this study is conserved between both species^6^. Rapid analysis and quantification of biological signatures allowed us to not only to describe significant bacterial intracellular changes pretomanid generates but crucially, distinguish its mode of action. The metabolite profiles were obtained using flow infusion electrospray ion high resolution mass spectrometry (FIE-HRMS) and the identification of compounds of interest was validated by matching retention times with standards, by gas-chromatography time-of flight mass spectrometry (GC-tofMS).

## Results and Discussion

### Pretomanid treated bacteria display a distinct metabolome

The metabolite profiles of mycobacteria cultures in mid-log phase were obtained at 0, 2, 4, 6 h following treatment with nine antibiotics: ampicillin, ethambutol, ethionamide, isoniazid, kanamycin, linezolid, rifampicin, streptomycin, pretomanid and an untreated control group. The concentration of antibiotic used was standardised in order to obtain 50 % of bacterial growth inhibition at 6 hours, as previously proposed^7^ (Table S1). Mass ions associated with a given antibiotic or its metabolites were eliminated from the data matrix prior to multivariate statistical analysis.

**Figure 1:**
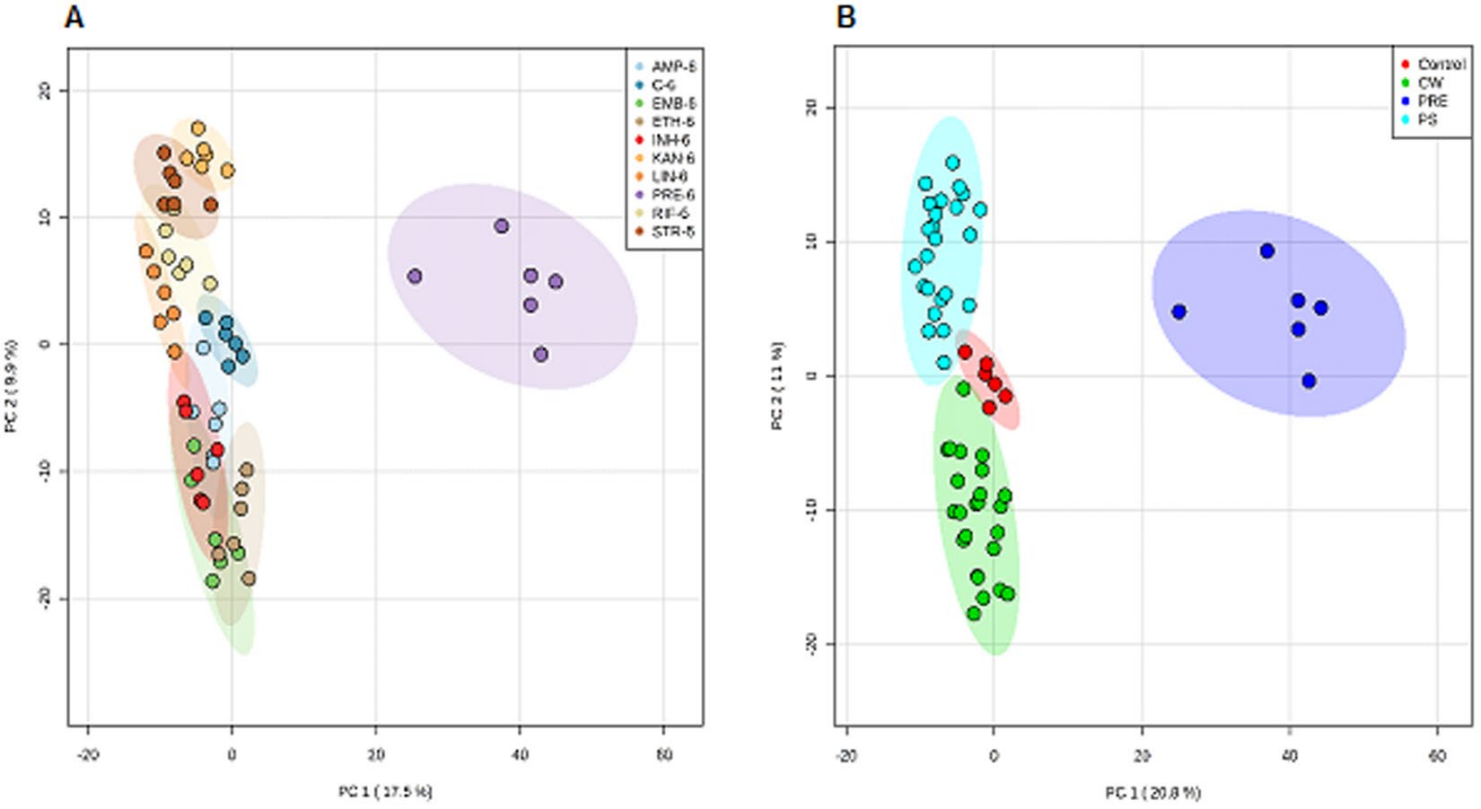
Demonstrative principal component analysis (PCA) score plots of FIE-MS data of 2285 normalised *m/z* intensity values (P < 0.05) in both negative and positive ionisation mode, 6 h after antibiotic treatment: (**A**) AMP-ampicillin, C-control group, EMB-ethambutol, ETH-ethionamide, INH-isoniazid, KAN-kanamycin, LIN-linezolid, PRE-pretomanid, RIF-rifampicin, STR-streptomycin; (**B**) samples were clustered based on their mechanism of action, CW-cell wall synthesis, PS-protein synthesis, PRE-pretomanid treatment. Coloured regions display 95% confidence.

Metabolomic similarities may expected in the profiles of bacteria treated with antibiotics targeting the same biological pathway. We observed such an effect using unsupervised principal component analysis (PCA) indicated distinctive clusters for antibiotics targeting different metabolic pathways (Fig. 1). For example, antibiotics targeting protein and cell wall biosynthesis pathways showed distinctive clustering patterns compared to others. Such observations confirm our assertion that untargeted metabolomics can to overcome the complexity and uncertainty of the available target discovery as a “stand-alone” methodology or via integration with other approaches, for example, transcriptomics. Crucially, bacteria treated with pretomanid displayed a different metabolic pattern compared to other antibiotics, suggesting that its mode of action was distinct. This significantly different metabolomic profile for pretomanid treatment was observed at all time-points (Fig. S1).

**Figure 2:**
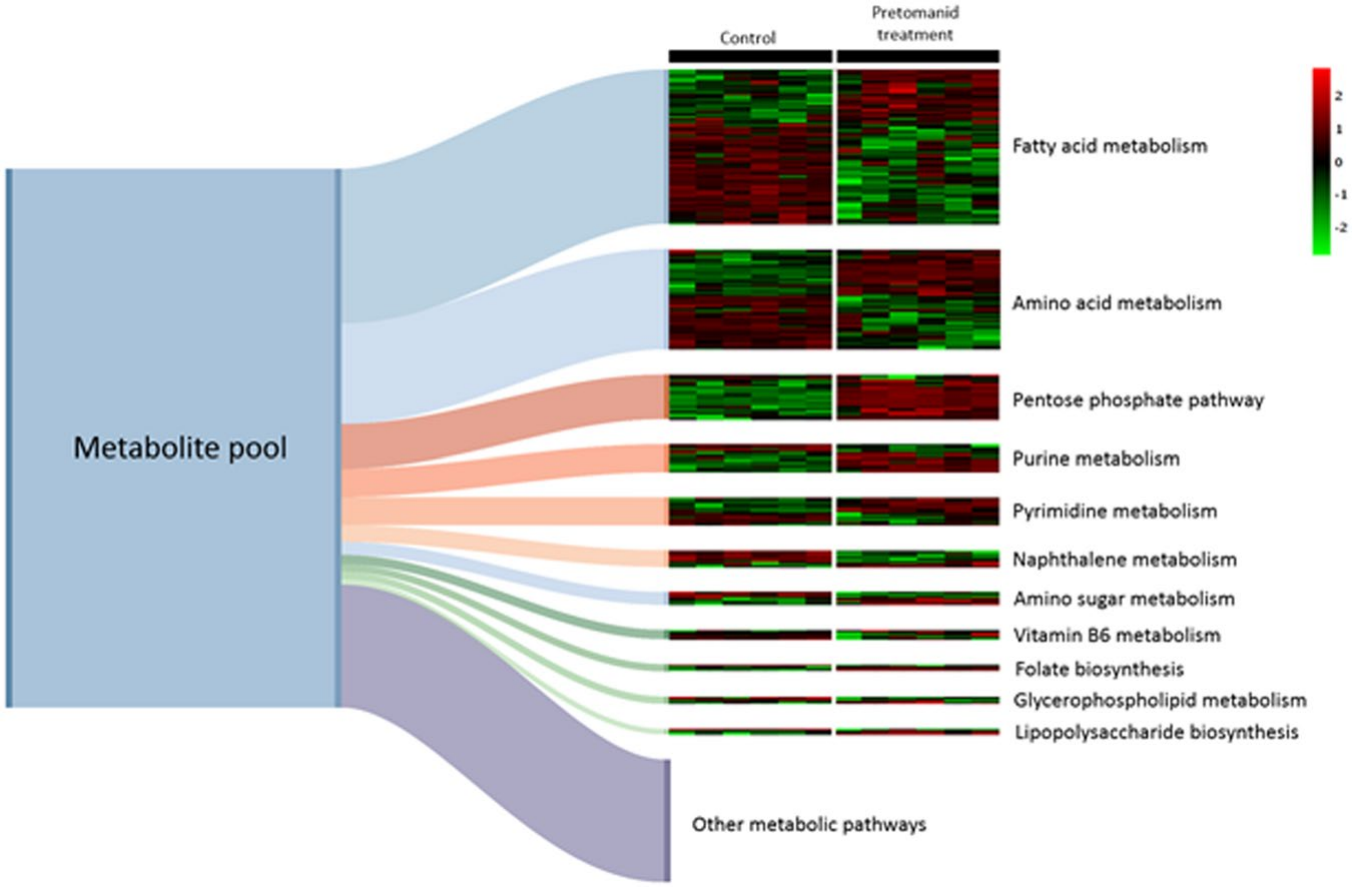
A pool of 794 putative identified metabolites that were significantly differed (P < 0.05) between samples from control and treatment with pretomanid were mapped on to specific metabolic pathways as defined by KEGG (Kyoto Encyclopaedia of Genes and Genomes). The Sankey diagram shows the relative accumulation of metabolites per pathway. Only pathways where more than three constituent metabolites have been targeted are depicted as discrete pathways. The associated heatmap schematically represents the relative concentration of each metabolite in six replicates of untreated controls and pretomanid-treated (t = 6 h) *Mycobacterium smegmatis* cultures.

All bacterial metabolites that significantly differed (P < 0.05) on pretomanid treatment compared to controls were identified. This allowed us to identify the most prominent changes associated to a specific metabolic pathway following mapped on to mycobacterial metabolism as defined by KEGG. Metabolites in fatty acid metabolism, amino acid metabolism, pentose phosphate pathway, purine metabolism and pyrimidine metabolism were found to significantly differ between control and pretomanid treatment (Fig. 2). In particular, metabolites from the pentose phosphate pathway accumulated on pretomanid treatment. No other major metabolic processes were shown to change in manners which would suggest a possible cell inhibitory mechanism. Mycolic acids were previously suggested as the pretomanid target^2^, and we also notes a large fraction of metabolites associated to fatty acid metabolic perturbation (Fig. 2). This could suggest a pretomanid mode of action based on altered mycolic acid synthesis. However, we propose alternative targets, given the distinctive PCA clusters shown in Fig. 1 which were, for example different from isoniazid, which inhibits mycolic acid synthesis^8^.

### Accumulation of phosphate sugars leads to increased levels of toxic methylglyoxal

**Figure 3:**
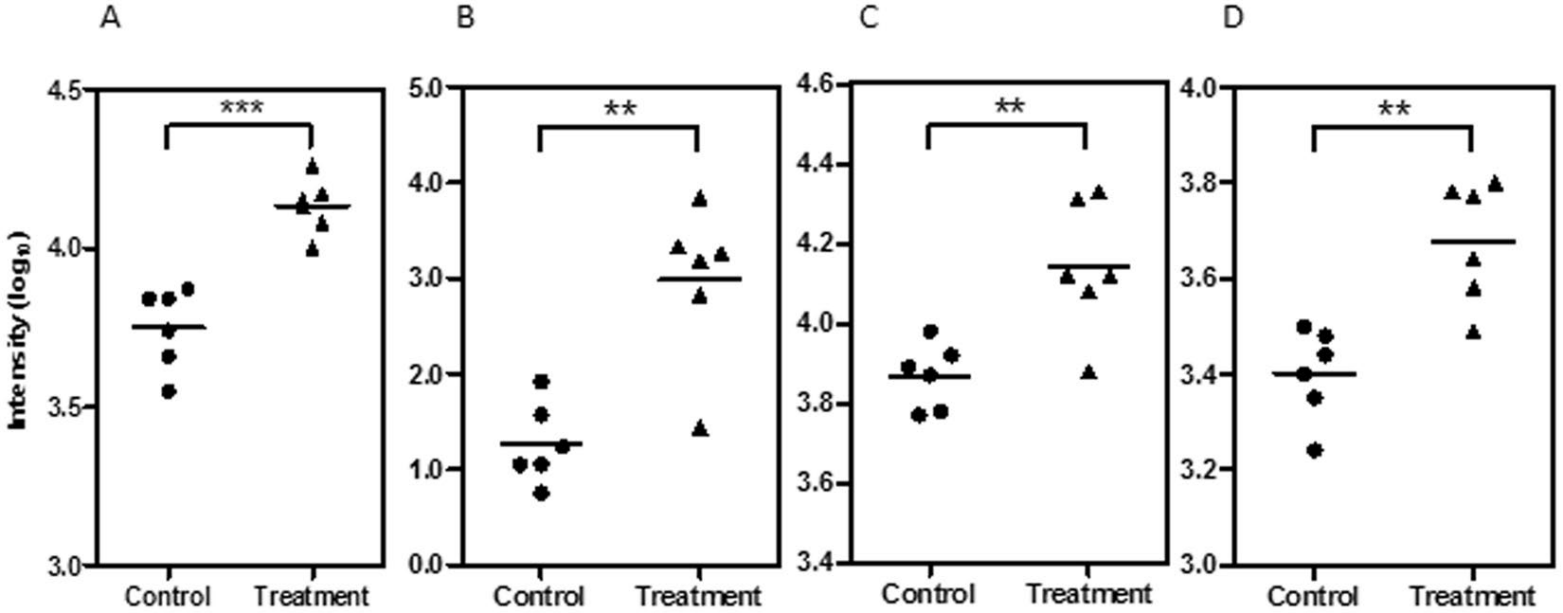
Accumulation of ribose-6-phosphate (**A**), glyceraldehyde-3-phosphate (**B**), fructose-6-phosphate (**C**) and methylglyoxal (**D**) at 6 h in *M. smegmatis* under pretomanid treatment (triangle) and control samples (circle). Log_10_ normalisation using logarithmic transformation of data. **P < 0.01, ***P < 0.001.

The analysis of rapid metabolic changes of bacteria following antibiotic treatment can occasionally be deceptive. These changes are adaptive and can be related to indirect effects of the drug that ultimately do not relate to cell death or cell division arrest mechanisms. To counter any false leads were targeted metabolites linked to crucial metabolic processes which were conserved across all mycobacterial species. Our metabolomic data indicated that pretomanid treatment led to the significant (P < 0.05) accumulation of metabolites downstream from glucose-6-phosphate in the pentose phosphate pathway. These included of ribose-5-phosphate, fructose-6-phosphate and glyceraldehyde-3-phosphate, particularly at 6 h after treatment with pretomanid (Fig. 3A–C). The identity of ribose-5-phosphate and fructose-6-phosphate were unequivocally confirmed by GC-tofMS through comparison to commercially available standards, with retention times of 7.37 and 7.93 min, respectively.

These observations align with the known mechanism of activation of the prodrug pretomanid in all mycobacteria. Activation of pretomanid is known to involve reduction by a deazaflavin dependent nitroreductase (Ddn). This conversion is catalysed by the cofactor F_420_H_2_ and the reductive form of F_420_ is linked to the conversion of glucose-6-phosphate (G6P) to 6-phosphoglucolactone by glucose-6-phosphate dehydrogenase (G6PD)^9–11^. The reaction is catalysed by F_420_-dependent glucose-6-phosphate dehydrogenase (FGD) leading to the biosynthesis of ribulose-5-phosphate in the pentose phosphate pathway, further yielding ribose-5-phosphate and xylulose-5-phosphate and later, fructose-6-phosphate and glyceraldehyde-3-phosphate. Since the activation of pretomanid is F_420_H_2_-dependent, the biosynthesis of this coenzyme is an absolute requirement for its activity. The only way to obtain this coenzyme is by the conversion of glucose-6-phosphate into 6-phosphoglucolactone, which will trigger an accumulation of downstream sugar phosphates.

The consequences of the accumulation of phosphate sugars, such as fructose-6-phosphate, have been studied in the context of bacterial metabolism in *E. coli*. The cessation of growth and loss of cell viability has been assumed to be due to the accumulation of methylglyoxal, which is produced as the result of the intracellular accumulation glucose-6-phosphate and fructose-6-phosphate^12,13^. This highly reactive aldehyde induces toxicity based on the interaction with proteins/amino acids and DNA/nucleotides, leading to the accumulation of glycated products and resultant cell arrest^14–16^. As a result, methylglyoxal intracellular production should be maintained within a certain range otherwise the bacteria’s electrophile protection mechanisms fail, leading to cell arrest^17^. This observation also aligns with the observations of Noy *et*. when examining the regulation of the glycolytic pathway in *M. tuberculosis*. Pyruvate kinase (encoded by *pykA*) catalyzes the rate-limiting step in glycolysis and *pykA*, mutated strains were not viable when grown on such as glucose. Analyses of the *pykA*, mutants showed that the shifts in metabolism included glucose-6-phosphate diversion toward ribulose-5-phosphate and the production of methylglyoxal^18^. Within a mammalian context, methylglyoxal is known to induce apoptosis in different cell lines by interfering with glutathione metabolism to hinder the neutralisation of oxidative radicals^19^.

**Figure 4:**
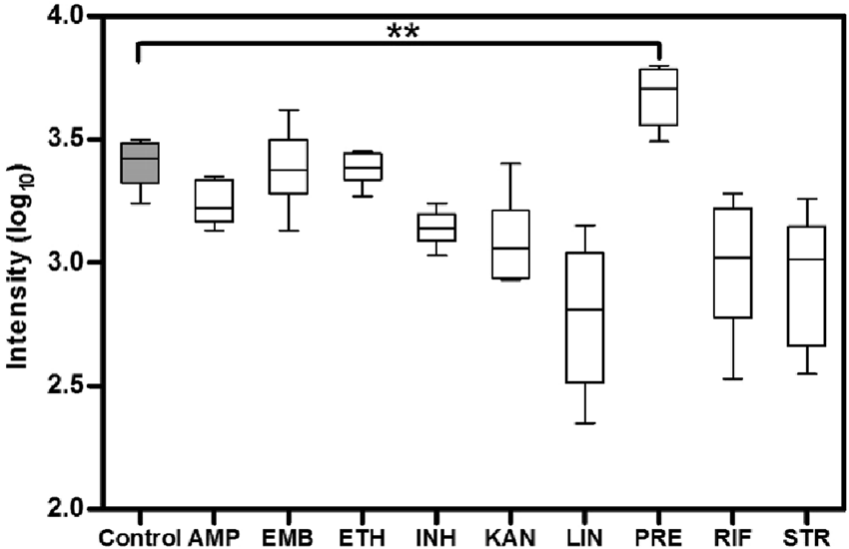
Accumulation of methylglyoxal in *M. smegmatis* is only visible under pretomanid treatment. AMP-ampicillin, EMB-ethambutol, ETH-ethionamide, INH-isoniazid, KAN-kanamycin, LIN-linezolid, PRE-pretomanid, RIF-rifampicin, STR-streptomycin. Log_10_ normalisation using logarithmic transformation of data. Data are means ± SD. **P < 0.01.

**Figure 5:**
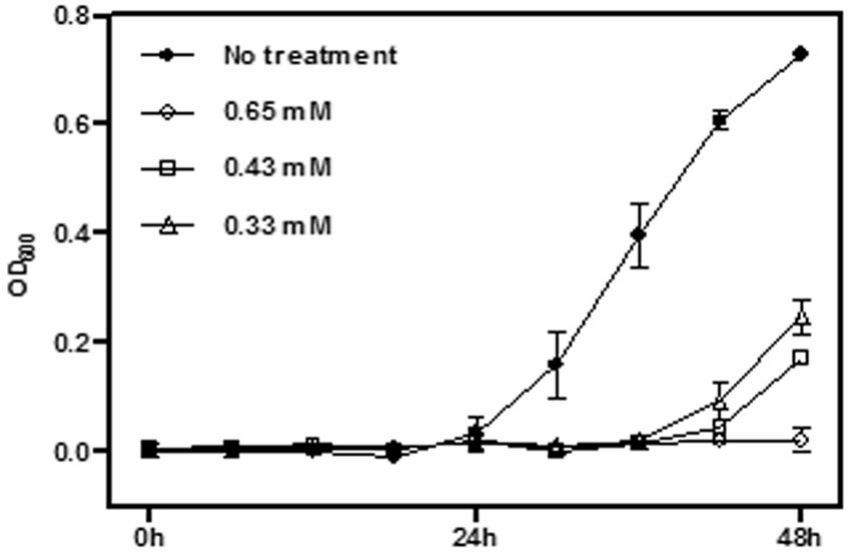
*M. smegmatis* growth inhibition by methylglyoxal, at 0.33, 0.43 and 0.65 mM, over 48 h.

**Figure 6:**
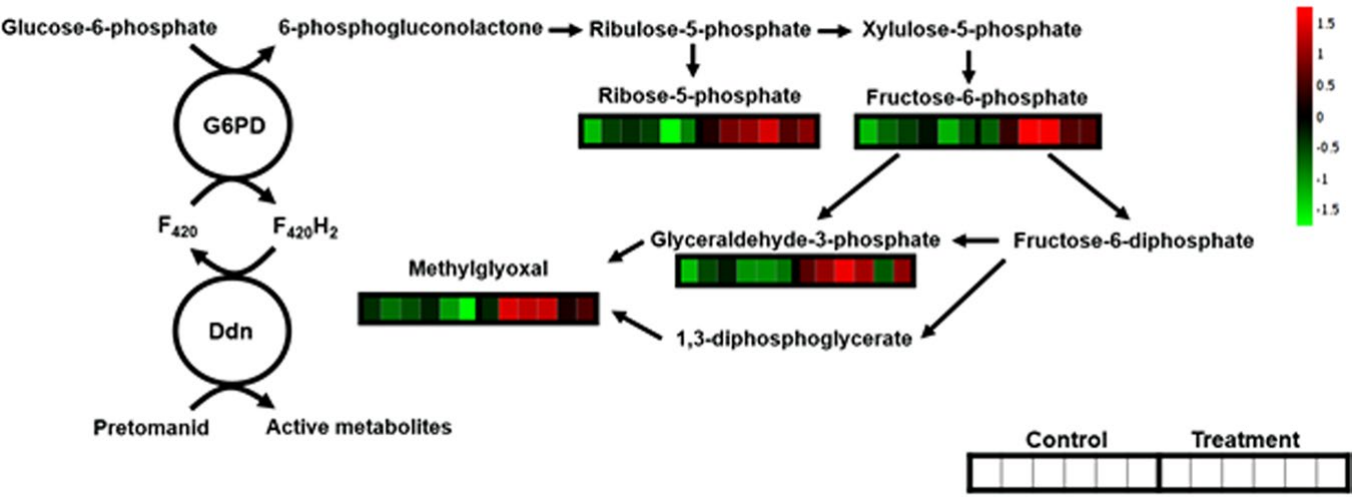
Pentose phosphate pathway. Pretomanid induces the conversion of glucose-6-phosphate in 6-phosphogluconolactone and consecutively the accumulation metabolites downstream the pentose phosphate pathway. G6PD - glucose-6-phosphate dehydrogenase, Ddn - deazaflavin dependent nitroreductase. The associated heatmap schematically depicts the relative concentration of each metabolite in six replicates of untreated controls and t = 6 h pretomanid-treated *Mycobacterium smegmatis* liquid cultures.

Given this, the accumulation of methylglyoxal that was detected at 6 h after pretomanid treatment (Fig. 3D) was a significant observation. We took pains to confirm the identity of the methylglyoxal ions in a follow-up experiment where FIE-HRMS was conducted with samples spiked with the commercially available standard (Fig. S2). The relative accumulation of methylglyoxal compared to controls was not seen when *M. smegmatis* is treated with any other antibiotic, confirming this as a unique mode of action of pretomanid (Fig. 4). To confirm the influence of methylglyoxal in mycobacterial growth, the MIC of this compound against *M. smegmatis* was measured (Fig. 5). Methylglyoxal was shown to be toxic at a MIC of 0.65 mM, being in line with previous results obtained against the pathogenic strain *M. tuberculosis*^13^. This modest MIC is likely to be due to difficulties in bacterial cell internalisation. Nevertheless, this result confirms the toxic effect of methylglyoxal in mycobacteria due to overloading the pentose phosphate pathways (Fig. 6). The importance of this metabolic pathway establishes that our observations in *M. smegmatis* will be of relevance to the slower growing *M. tuberculosis*. Further, the known effects of methylglyoxal accumulation^14–16^ is likely to explain the wider effects on metabolite changes in amino acid, purine and pyrimidine metabolism (Fig. 2), The increased levels of methylglyoxal would only have repercussions in bacterial cells, since the enzyme Ddn is only present in *Mycobacterium* and other Actinobacteria, except *M. leprae*^20^. This further suggests, to a certain extent, the safety of pretomanid for human use. Clearly, our conclusions needs to be substantiated by *in vivo* studies based on such as macrophage infection models where the effects of for example, hypoxia on methylgloxal accumulation can be assessed.

### High resolution metabolomic profiling; a new approach for drug mode of action studies

Using robust and reproducible information has allowed us to comprehend the mechanism of action of pretomanid against *Mycobacterium*. By applying metabolomics we were able to show that pretomanid has a distinctive mode of action different from other currently known antibiotics. We have also gained insights into its specific mode of action, disrupting normal cell sugar phosphate metabolism leading to the accumulation of toxic methylglyoxal. This proposed mechanism would be effective against both replicative and latent *Mycobacteria*. Henceforth, it is expected these conclusions will provide deeper insights not only into the metabolism of mycobacterial strains resistant to pretomanid, but also in the use of the discovered biosynthetic pathway, the pentose phosphate’s accumulation and in the rational development of new anti-tubercular molecules.

## Materials and Methods

### Chemicals

Pretomanid, streptomycin sulfate, ethambutol dihydrochloride, ethionamide, isoniazid, linezolid and standards (glucose-6-phosphate, ribose-5-phosphate, fructose-6-phosphate and methylglyoxal) were obtained from Sigma. Kanamycin sulfate, ampicillin sodium salt and rifampicin were obtained from Gibco, Melford and Duchefa Biochemie respectively.

### Bacterial growth conditions

All the procedures were carried out in a biosafety containment level 2. *Mycobacterium smegmatis* strain was purchased from National Health England (ATCC 19420) and used for testing all the compounds. *M. smegmatis*, rapidly growing mycobacteria, was cultured in Luria-Bertani (LB) medium supplemented with 0.2% (v/v) glycerol and 0.05% (v/v) of Tween 80 at 37 °C with aeration at 200 rpm until OD_600_ 0.6 was reached.

### Determination of *in vitro* antibacterial activity and dosage for metabolomics

The minimum inhibitory concentration (MIC) of methylglyoxal was determined using broth microdilution method, in 96-well plate format, in fresh LB medium. All samples were tested in triplicate in an initial bacterial concentration of 5.0 × 10^5^ CFU/mL. The MIC was determined as the lowest concentration of a drug at which no growth was visible after 48 h. The optical density OD_600_ was measured in a Hidex plate spectrophotometer.

To access the concentration of antibiotics required to inhibit the bacterial growth by 50%, a microdilution of all antibiotics in a bacterial culture of OD_600_ 0.6 was performed. The optical density OD_600_ was measured after 6 h of treatment. The dosage of antibiotic to use in the metabolomic protocol was then calculated.

### Sample preparation and metabolite extraction for FIE-HRMS and GC-tofMS

The bacterial incubation throughout the sampling consisted in constant shaking at 200 rpm at 37 °C. Six biological replicates of each isolate treated with antibiotic and the untreated control group were independently cultured.

All samples were collected during mid-exponential growth phase. A 10 mL aliquot of bacterial culture (OD_600_ at time 0 h was 0.6) was harvested at 0, 2, 4 and 6 h after the treatment with the respective antibiotic. The samples were stored at −80 °C after the cellular metabolism was rapidly quenched with liquid N_2_. After thawing, the samples were centrifuged at 10 °C at 4500 rpm. The yielded pellet was then washed with 10 mL of cold saline solution (0.85 % NaCl) with consequent centrifugation at 10 °C at 4500 rpm. All samples were adjusted to an OD_600_ of 1 before the extraction with 200 μL of a chloroform/methanol/water (1:3:1) solution. The extraction was achieved by four freeze-thaw cycles with periodic vortexing. After a final centrifugation at 4500 rpm, 170 μL of the particle-free supernatant was transferred into a microcentrifuge tube. An additional extraction with 180 of chloroform/methanol/water (1:3:1) was done and the new supernatant, after final centrifugation, was combined with the supernatant from the first extraction. From this mixture, 50 μL were transferred into a HPLC vial containing a 0.2 mL flat bottom micro insert for FIE-HRMS analysis; the remaining volume (~120 μL) was transferred into a 1.5 mL microcentrifuge tube and evaporated under vacuum (UniEquip UNIVAPO 150 H, UK) for further GC-tofMS analysis.

### Metabolite profiling by GC-tofMS analysis

For gas chromatography time-of-flight mass spectrometry (GC-tofMS) dried samples were derivatized first by methoximation using 5 μL of a 20 mg mL^−1^ solution of methoxyamine hydrochloride (Fluka) in pyridine (Fluka) at 30 °C for 90 min to protect carbonyl moieties. Acidic protons were subsequently derivatized with 10 μL N-methyl-N-trimethylsilyltrifluoride (MSTFA, M&N) at 37 °C for 30 min. Derivatized samples were transferred into 200 μL glass vials (Chromacol) and 1 μL was injected split-less into a Leco Pegasus III GC-tofMS system (St. Joseph, USA) equipped with an Agilent 6890 N gas chromatograph equipped with a 20 m DB5-MS column (0.25 mm ID, 0.25μm film) and a Focus autosampler (Anatune, UK). Injector temperature was 250 °C, the interface was set to 260 °C and the ion source temperature held at 230 °C. Helium flow was 1.4 mL min^−1^. After 1 min at 80 °C, oven temperature was increased by 30 °C min^−1^ to 330 °C, held at 330 °C for 3 min and cooled to 80 °C. Automated deconvolution and peak finding was performed using ChromaTof software (Leco, St. Joseph, USA) and peak alignment was carried out in MATLAB (V6.5.1, The MathWorks).

### Metabolite fingerprinting by flow injection electrospray high resolution mass spectrometry (FIE-HRMS)

Flow infusion electrospray high-resolution mass spectrometry (FIE-HRMS) was performed using an Exactive HCD mass analyser equipped with an Accela UHPLC system (Thermo-Scientific) which generated metabolite fingerprints in both, positive and negative ionisation mode, in a single run. Samples (20 µL volume) were injected into a flow of 100 µl.min^−1^ methanol: water (70:30, v/v). Ion intensities were acquired between *m/z* 50 and 1000 for 3.5 min at a resolution setting of 100,000 (at *m/z* 200) resulting in 3 ( ± 1) ppm mass accuracy. ESI source parameters were set according to manufacturer’s recommendations. Raw files were exported to CDF-files, mass aligned and centroided in MATLAB (V8.2.0, The MathWorks) maintaining highest mass accuracy. Mass spectra around the apex of the infusion peak were combined in a single intensity matrix (runs x *m/z*) for each ion mode. Data from intensity matrix was log-transformed before further statistical analysis.

### Metabolomic data analysis

Univariate and multivariate analyses were performed with MetaboAnalyst 3.0. Differences in the metabolomics profiles of samples were analysed with unsupervised PCA (principal component analysis) and supervised PLS-DA (partial least-squares discriminant analysis). The significance of the cross-validated P-values, based on one-way analysis of variance (ANOVA), was set to P < 0.05. Multiple comparison and *post hoc* analysis used Tukey’s Honestly Significant Difference (Tukey’s HSD). Both ANOVA and Tuckey’s HSD allowed the identification of significant metabolite changes between groups (control or antibiotics). Metabolites that did not show significant differences between treatment and control were not further analysed. For each mass,-ion (*m/z*) the annotation was made using a 3 ppm tolerance on their accurate mass. Metabolomic annotation was made using MZedDB^21^ (http://maltese.dbs.aber.ac.uk:8888/hrmet/index.html), the *E. coli* Metabolome Database (ECMDB, http://ecmdb.ca/) and LipidMaps (http://www.lipidmaps.org/) databases, considering the following possible adducts: [M + H] + , [2 M + H] + , [M + 2 H]2 + , [M + 3 H] + , [M + Na] + , [M + Na + 2 H] + , [2 M + Na] + , [M + 2Na−H] + , [M + 2Na + H] + , [M + Na + H]2 + , [M + 2Na] + , [M + 3Na]3 + , [M + K] + , [M + K + H]2 + , [M + 2 K + H] + , [M−FA + H] + , [M−H_2_O + H] + , [2 M + 3H_2_O + 2 H]2 + , [M + NH4] + , [M + NH4 + H] + , [M + NH4 + H]2 + , [2 M + NH4] + , [M + ACN + H] + , [M + ACN + Na] + , [2 M + ACN + H] + , [M + ACN + 2 H] + , [M + 2ACN]2 + , [M + 2ACN + H] + , [M + 2ACN + 2 H] + , [M + 3ACN + 2 H] + , [M + ACN + Na] + , [M + CH3OH + H] + ; [M−H]−, [2M−H]−, [3M−H]−, [M−2H]−, [M−3H]−, [M−H_2_O−H]−, [M + Cl]−, [M + FA−H]−, [2 M + FA−H]−, [M + Hac−H]−, [M + Na−2H]−, [2 M + Na−2H]−, [M + K−2H]−, [M + TFA−H]−. The targeted metabolites were mapped on to Kyoto Encyclopedia of Genes and Genomes (KEGG) for pathway analysis (http://www.genome.jp/kegg/pathway.html).

For definitive metabolite identification, the retention time of standards (glucose-6-phopshate, ribose-5-phosphate and fructose-6-phosphate) was analysed by gas chromatography time-of-flight mass spectrometry. Methylglyoxal was identified by overlay of spiked samples injected in FIE-MS; the tolerance on the accurate mass for the peak annotation was 1 ppm.

## Electronic supplementary material

Supporting Information

Supplementary Dataset 1

## References

[1] WHO. 2016 Global tuberculosis report. (WHO Press, 2016).

[2] Stover CK (2000). A small-molecule nitroimidazopyran drug candidate for the treatment of tuberculosis. Nature.

[3] Singh R (2008). PA-824 kills nonreplicating Mycobacterium tuberculosis by intracellular NO release. Science.

[4] Dogra M (2011). Comparative bioactivation of the novel anti-tuberculosis agent PA-824 in Mycobacteria and a subcellular fraction of human liver. Br. J. Pharmacol..

[5] Overy DP (2008). Explanatory signal interpretation and metabolite identification strategies for nominal mass FIE-MS metabolite fingerprints. Nat. Protoc..

[6] Baloni P, Padiadpu J, Singh A, Gupta KR, Chandra N (2014). Identifying feasible metabolic routes in Mycobacterium smegmatis and possible alterations under diverse nutrient conditions. BMC Microbiol..

[7] Halouska S, Fenton RJ, Barletta RG, Powers R (2012). Predicting the *in Vivo* Mechanism of Action for Drug Leads Using NMR Metabolomics. ACS Chem. Biol..

[8] Timmins GS, Deretic V (2006). Mechanisms of action of isoniazid. Mol. Microbiol..

[9] Manjunatha UH (2006). Identification of a nitroimidazo-oxazine-specific protein involved in PA-824 resistance in Mycobacterium tuberculosis. Proc. Natl. Acad. Sci. United States Am..

[10] Bashiri G, Squire CJ, Moreland NJ, Baker EN (2008). Crystal structures of F420-dependent glucose-6-phosphate dehydrogenase FGD1 involved in the activation of the anti-tuberculosis drug candidate PA-824 reveal the basis of coenzyme and substrate binding. J. Biol. Chem..

[11] Cellitti SE (2012). Structure of Ddn, the deazaflavin-dependent nitroreductase from Mycobacterium tuberculosis involved in bioreductive activation of PA-824. Structure.

[12] Kadner RJ, Murphy GP, Stephens CM (1992). Two mechanisms for growth inhibition by elevated transport of sugar phosphates in Escherichia coli. J. Gen. Microbiol..

[13] Pethe K (2010). A chemical genetic screen in Mycobacterium tuberculosis identifies carbon-source-dependent growth inhibitors devoid of *in vivo* efficacy. Nature Communications.

[14] Thornalley PJ (2003). Quantitative screening of advanced glycation endproducts in cellular and extracellular proteins by tandem mass spectrometry. Biochemical Journal.

[15] Thornalley PJ (2010). Imidazopurinones are markers of physiological genomic damage linked to DNA instability and glyoxalase 1-associated tumour multidrug resistance. Nucleic Acids Res..

[16] Murata-Kamiya N, Kamiya H (2001). Methylglyoxal, an endogenous aldehyde, crosslinks DNA polymerase and the substrate DNA. Nucleic Acids Res..

[17] Booth IR (2003). Bacterial production of methylglyoxal: a survival strategy or death by misadventure?. Biochem. Soc. Trans..

[18] Noy T (2016). Central role of pyruvate kinase in carbon co-catabolism of *Mycobacterium* t*uberculosis*. J, Biol. Chem..

[19] Rachman H (2006). Critical role of methylglyoxal and AGE in mycobacteria-induced macrophage apoptosis and activation. PLoS One.

[20] Manjunatha UH (2006). Mycobacterium leprae Is Naturally Resistant to PA-824. Antimicrobial Agents and Chemotherapy.

[21] Draper J (2009). Metabolite signal identification in accurate mass metabolomics data with MZedDB, an interactive m/z annotation tool utilising predicted ionisation behaviour ‘rules’. BMC Bioinformatics.

