# Supplementary material for: Untargeted metabolomics reveals a new mode of action of pretomanid (PA-824)
Source: Sci Rep. 2018 Mar 23;8:5084. doi: 10.1038/s41598-018-23110-1 (PMC5865180; doi:10.1038/s41598-018-23110-1)
Supplement: Supplementary file 1 — Supporting Information [file 41598_2018_23110_MOESM1_ESM.doc]

Untargeted metabolomics reveals a new mode of action of pretomanid (PA-824)

Rafael Baptista^1^, David M. Fazakerley^1^, Manfred Beckman^1^, Les Baillie^2^, Luis A. J. Mur^1*^

1 Institute of Biological, Environmental and Rural Sciences, Aberystwyth University, Penglais Campus, Aberystwyth, Wales, UK, SY23 2DA

2 School of Pharmacy and Pharmaceutical Sciences, Cardiff University, Redwood Building, Cardiff, Wales, UK, CF10 3NB

*email: lum@aber.ac.uk

**Supporting Information**

**1. Supplementary tables**

Table S1 – Dosages used in the treatment with antibiotics, against *Mycobacterium smegmatis.*

|  | Dosage (μg/mL) |
| --- | --- |
| Ampicillin | 250.0 |
| Ethambutol | 31.25 |
| Ethionamide | 250.0 |
| Isoniazid | 31.25 |
| Kanamycin | 1.328 |
| Linezolid | 3.963 |
| Pretomanid | 21.25 |
| Rifampicin | 3.963 |
| Streptomycin | 1.328 |

**2. Supplementary figures**


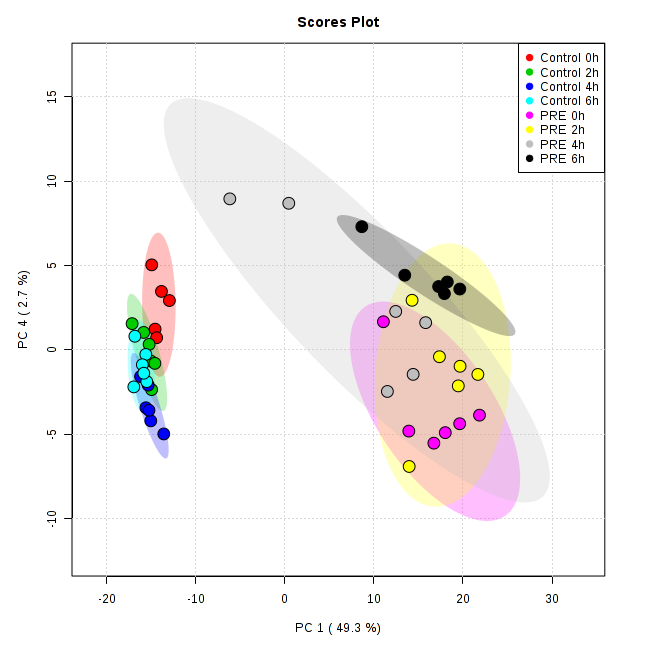


Fig. S1. Principal component analysis (PCA) score plots of FIE-MS data of 1122 normalised *m/z* intensity values (P<0.05) in both negative and positive ionisation mode, compare treatment by pretomanid (PRE) and control by time-point. Coloured areas display 95% confidence regions.


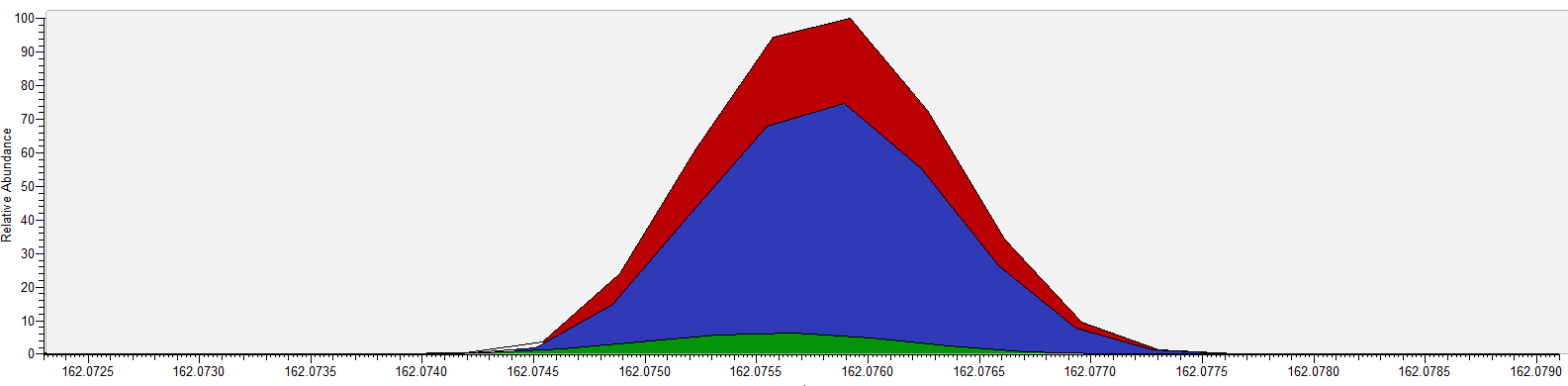


Fig. S2. FIE-HRMS spectra of methylglyoxal relative intensity peak *m/z* 162.076040, identified as the [2M+NH4]+ adduct. Overlay of methylglyoxal standard (red) with sample spiked with the standard (blue) and sample peak (green). The tolerance on their accurate mass for the peak annotation was 1 ppm.
